# A novel *SoxB2* gene is required for maturation of sperm nucleus during spermiogenesis in the Chinese mitten crab, *Eriocheir sinensis*

**DOI:** 10.1038/srep32139

**Published:** 2016-08-26

**Authors:** Zhi-Qiang Liu, Xue-Hui Jiang, Hai-Yan Qi, Liang-Wei Xiong, Gao-Feng Qiu

**Affiliations:** 1 Key Laboratory of Exploration and Utilization of Aquatic Genetic Resources Certificated by Ministry of Education, College of Fisheries and Life Science, Shanghai Ocean University, 999 Hucheng Huan Road, Shanghai, 201306, P. R. China

## Abstract

*SRY*-related HMG box (*Sox*) genes are characterized by the presence of a DNA-binding HMG domain and involved in a diverse range of developmental processes. In this study, we identified a novel *Sox* gene, designated as *EsSoxB2-1*, from the Chinese mitten crab *Eriocheir sinensis*. The *EsSoxB2-1* encodes a protein of 259 amino acids, sharing the highest identity with the beetle *Tribolium castaneum* SOX21b. Unlike insect *Sox21b*, however, *EsSoxB2-1* is intronless and exhibits a gonad-specific expression pattern at both mRNA and protein level. Two core promoters in 5′ flanking region were demonstrated to be essential for inducing transcriptional regulatory activity. The transcription of *EsSoxB2-1* mRNA begins in spermatogonia stage, while the translation of *Es*SOXB2-1 protein initiates at spermiogenesis stage. Interestingly, *Es*SOXB2-1 protein was exclusively localized in the nucleus of spermatid and spermatozoa even at the end of acrosome reaction, and was bound to the uncondensed chromatin in nucleoplasm of mature spermatozoa. Knockdown of *EsSoxB2-1* by RNAi leads to abnormal transformation of the nucleus during spermiogenesis. Together, these findings demonstrated the requirement of *EsSoxB2-1* for the spermatozoa nucleus maturation and also suggested that *EsSoxB2-1* would be delivered into fertilized eggs along with chromatins as a paternal transcription factor for regulating early embryonic development.

*SRY* was first discovered in human Y chromosome as a sex-determining factor, and possesses a conserved high-mobility group (HMG) box^1^,^2^. The HMG box contains 79 amino acids and is responsible for binding the minor groove of DNA at the site of specific target sequences, (A/T)(A/T)CAA(A/T)G^3^,^4^, to regulate transcription of downstream genes in the sex-determining cascade by altering chromatin structure^5^,^6^. Although *SRY* homologues are found only in placental mammals and marsupials and are not evolutionally conserved in the animal kingdom^2^,^7^, a large *SRY*-related HMG box (*Sox*) gene family of transcription factors was subsequently identified in both vertebrates and invertebrates^2^,^8^,^9^. The members of *Sox* family play pivotal roles in cell differentiation and embryonic organogenesis including gonadogenesis, neurogenesis, chondrogenesis, and oligodendrocyte development^10^,^11^,^12^. To date, over 30 members have been isolated and classified into ten groups (A-J) mainly based on their sequences similarity of HMG box. These groups are: A, *SRY*/*Sry*; B, *Sox1*, *Sox2*, *Sox3*, *Sox14* and *Sox21*; C, *Sox4*, *Sox11*, *Sox12*, *Sox22* and *Sox24*; D, *Sox5*, *Sox6*, *Sox13* and *Sox23*; E, *Sox8*, *Sox9*, and *Sox10*; F, *Sox7*, *Sox17*, and *Sox18*; G, *Sox15* and *Sox20*; H, *Sox30*; I, *Sox31*; J, *Sox J.* Members of the same group usually share over 70% amino acids identity both within and outside the HMG domains^2^. Although all five members of the group B genes are intronless in vertebrates, sequence analysis and functional studies suggested that the group B *Sox* genes can be subdivided into two further groups; B1; *Sox1*, *Sox2* and *Sox3*; and B2; *Sox14* and *Sox21*^13^. The three subgroup B1 members act as transcriptional activators, while the B2 members (SOX14 and -21) are transcriptional repressors^13^. In insects, four group B genes, *SoxNeuro (SoxN*), *Dichaete*, *Sox21a* and *Sox21b*, have been identified so far^2^. *SoxN* is associated with group B1 and the latter three are physically linked in the genome and assigned to group B2. However, *SoxN* and *Dichaete* are intronless while *Sox21a* and *Sox21b* bear introns^14^.

Gonad or sex differentiation is the complicated differentiation models of organogenesis entailing biochemical and morphological reconstruction of the germ cell. A number of *Sox* genes have been implicated in these complicated procedures^15^,^16^,^17^. Besides *Sry* acting as a master sex determination gene in mammals, some other *Sox* genes are involved in sex differentiation and germ cell development based on the data of their expression profiles. In mice, *Sox3* is located on X chromosome and is regarded as the ancestor of *Sry*^7^. *Sox*3 is not required for sex determination, but is needed for normal gametogenesis such as male testis differentiation^17^. Targeted deletion of *Sox3* caused abnormal development of spermatogonia^16^. *Sox9* is expressed shortly after *Sry* in pre-Sertoli cells and was confirmed to be the only target of SRY in mediating a switch from the ovarian pathway to the testicular pathway^12^,^18^. And the specific expression of *Sox30* in the normal testes, but not in the germ cell-deficient testes, suggesting the involvement of *Sox30* in the differentiation of mouse male germ cells^19^. Despite *Sox* genes have been cloned and characterized in a wide variety of taxonomic groups, rare molecular data of *Sox* genes has been documented in crustaceans so far. In the present study, we identified a novel *Sox* gene, termed *EsSoxB2-1*, which displayed gonad-specific expression in the Chinese mitten crab, *Eriocheir sinensis*. The *Es*SOXB2-1 protein was exclusively localized in the nucleus of spermiogenic germ cells and was revealed to be involvement in sperm nucleus maturation.

## Results

### Full-length sequence of the crab *EsSoxB2-1* cDNA

**Figure 1:**
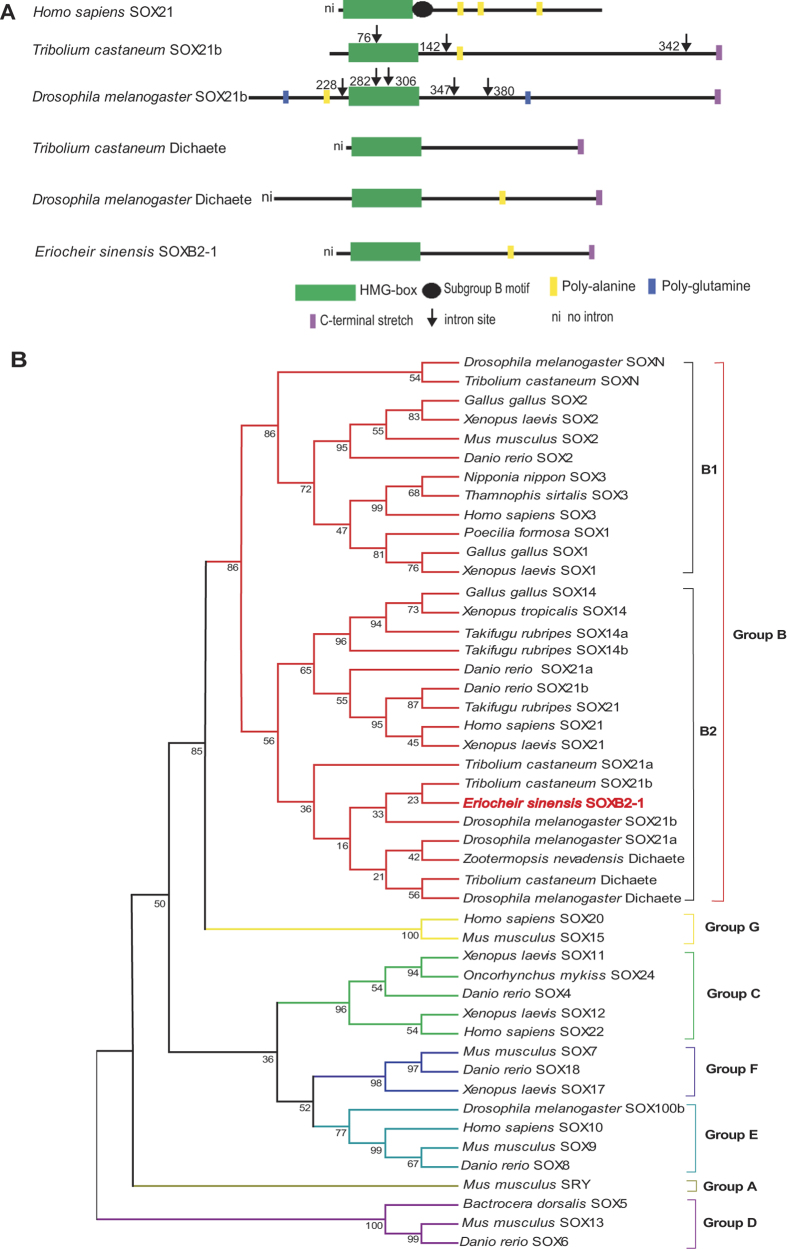
(**A**) Schematic representation of the mitten crab *Es*SOXB2-1 and other species SOXB proteins. (**B**) An unrooted phylogenetic tree generated by the NJ method using the multiple alignments of complete protein sequences of the mitten crab *EsSoxB2-1* and other species *Sox* genes. Numbers on each node are the bootstrap confidence values (%) in one thousand runs. The crab *Es*SOXB2-1 was highlighted by red font. The GenBank accession numbers are listed in Supplementary Table S1.

A cDNA fragment of 220 bp was amplified from the ovary by degenerate RT-PCR. The amplified fragment contained a conserved HMG box and was shown to be a *Sox* homologue by Blast analysis. Then the full-length cDNA of this *Sox* homologue was generated by 5′ and 3′ RACE. This cDNA is 951 bp in length and contains a 5′ untranslated region (UTR) of 54 bp, a open reading frame (ORF) of 777 bp, and a 3′UTR of 111 bp with a poly(A) tail. The ORF encodes a polypeptide of 259 amino acids (aa) with a predicted molecular weight of 28.55 KD. Amino acid sequence alignment showed the HMG box of the crab *Sox* homolog the highest (95%) identity with those of *Tribolium castaneum* SOX21b, but there is little (about 36%) sequence similarity outside the HMG box between them. The HMG box contains a consensus sequence RPMNAFMVW and four histidine residues (22, 49, 83, and 87aa) that are thought to be essential for the DNA-binding properties (Supplementary Figure S1). The crab *Sox* homolog also contains two nuclear localization signals (NLS)^20^ (K_24_RPMNAFMVWSRMQRRK_40_ and R_93_PRRKPKT_100)_, a nuclear export-signal (NES)^21^ and a small hydrophobic leucine-rich motif (ISKRLGSEWKLL), but lack a subgroup B motif (Fig. 1A), which appears next to the HMG box in vertebrate *Sox* B group^22^. Unlike *Tribolium castaneum* SOX21b, the crab *Sox* homolog contained one poly-alanine stretches at the carboxyl terminus^23^ (Fig. 1A). Accordingly, we designated this novel *Sox* homolog as *EsSoxB2-1*.

### Phylogenetic analysis

In an effort to determine the phylogenetic affinities between the crab *EsSoxB2-1* and other members of *Sox* family, an unrooted phylogenetic tree was constructed by the NJ method using the multialignment of complete protein sequences of various metazoans *Sox* including *Mus musculus* SRY^7^. As shown in Fig. 1B, the previously established groupings of *Sox* are supported by the tree^2^. Various *Sox* groups were assigned into different clades. The crab *EsSoxB2-1* falls into *Sox*B group and was first clustered with the beetle *Tribolium castaneum* and the fruit fly *Drosophila melanogaster* SOX21b, and then they were together clustered with SOX21a and Dichaete in *Sox* B2 group, although with less confidence.

### Gene structure and promoter activity of the crab *EsSoxB2-1*

The genomic sequence of *EsSoxB2-1* gene was amplified by PCR using a pair of gene-specific primers set at the 3′ and 5′ ends of the cDNA sequence. The retrieved genomic sequence was in excellent accord with its corresponding cDNA sequence (Supplementary Figure S1), indicating that the novel *EsSoxB2-1* gene contains no intron.

The 5′-flanking sequence of *EsSoxB2-1* gene was obtained by genome walking method. Sequence analysis showed that the 1509 bp 5′-flanking region contained two basal core promoters (−628/−577 bp and −493/−444 bp) and a 377 bp CpG island (−299/+78 bp). Many potential transcription factor binding sites were identified in the region from the transcription start site (TSS) to the core promoters. Some of binding sites were given more attention such as SRY/SOX, CATA, GATA-1, E2F, CREB and TATA (Supplementary Figure S2).

**Figure 2:**
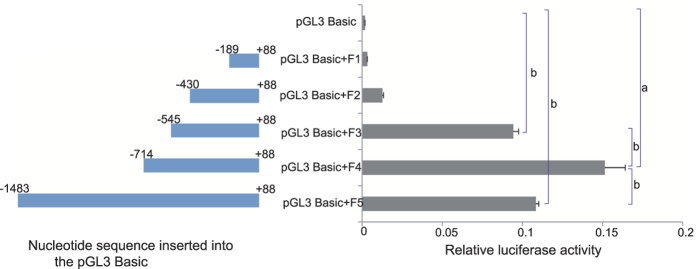
Transcriptional activity analysis of 5′ flanking regions of *EsSoxB2-1* gene in pGL3 Basic vector containing dual-luciferase reporter genes. F1, F2, F3, F4 and F5 represent various lengths of 5′-flanking sequences as shown in supplementary Figure S2. The relative activity is the mean value of three repetitions. ^a^*p* < 0.01, ^b^*p* < 0.05.

The promoter activity in 5′ -flanking region of the *EsSoxB2-1* gene was assayed using Dual-Luciferase Reporter Assay System (Promega). To determine which fragment within the 5′-flanking region responsible for transcriptional regulatory activity, various lengths of the 5′-flanking sequences F1 (−189/+88 bp), F2 (−430/+88 bp), F3 (−545/+88 bp), F4 (−714/+88 bp) and F5(−1483/+88 bp) were cloned into the promoterless pGL3-Basic plasmid containing luciferase genes, respectively (Supplementary Figure S2). Significantly high activities were detected in F3, F4, and F5 (Fig. 2). The promoter activity of F4 (−714/+88 bp) was much higher than that of the full length of the 5′-flanking region F5 (−1483/+88 bp), indicating the existence of silencing sequence elements within the fragment from −1483 to −714 bp. When excluding one core promoter (−628/−577 bp), the promoter activity of F3 displayed significant reduction compared to F4 (−714 to + 88) (Fig. 2). When excluding both of the two core promoters (−628/−577 bp and −493/−444 bp), the promoter activity of F2 (−430/+88 bp) and F1 (−189/+88 bp) became extremely low similar with the pGL3 Basic empty plasmid (Fig. 2). These data demonstrated that the two core promoters (−628/−577 bp) are essential for inducing transcriptional regulatory activity.

### Tissue distribution of *EsSoxB2-1* mRNA and *Es*SOXB2-1 protein

**Figure 3:**
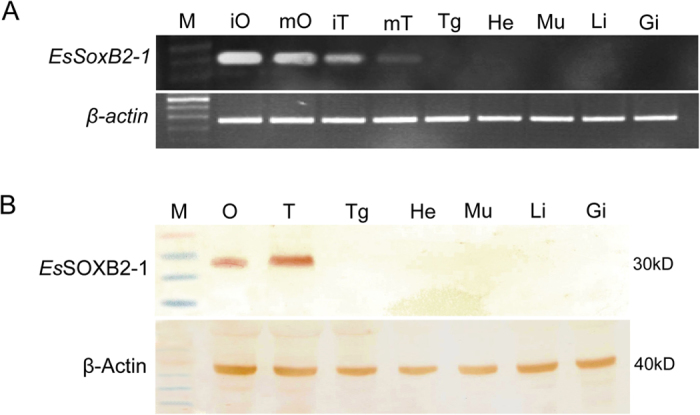
RT-PCR analysis (**A**) of tissue distribution of the crab *EsSoxB2-1* mRNA using *β*-actin as an internal reference and Western blot analysis (**B**) of tissue distribution of the crab *Es*SOXB2-1 protein using rabbit anti-*Es*SOXB2-1 protein as first antibodies. M, molecular weight standards; iO, immature ovay; mO, mature ovary; iT, immature testis; mT, mature testis; Tg, thoracic ganglion; He, heart; Mu, muscle; Li, liver; Gi, gill; T, testis; O, ovary.

Interestingly, RT-PCR analysis of seven types of adult tissues showed that *EsSoxB2-1* was exclusively expressed in testis and ovary, but not found in any other somatic tissues examined. The amount of *EsSoxB2-1* transcripts is higher in ovary than in testis, with especially higher in immature ovary and testis. As an internal reference, the *β*-actin was simultaneously amplified using the same cDNA samples, and similar amounts of amplified product were obtained from all of the tissues (Fig. 3A).

Western blot analysis was performed by using the rabbit anti-*Es*SOXB2-1 serum. A specific target band of about 30KD protein was detected in the testis and ovary (Fig. 3B), while no signal was seen in the negative control using the normal (preimmune) rabbit serum instead of the primary specific antibody (data no shown). As a loading control, β-Actin protein was detected in all the tissues examined. These data demonstrated that *Es*SOXB2-1 protein also displayed specific expression in the testis and ovary. However, unlike mRNAs, the *Es*SOXB2-1 protein is expressed much higher in testis than in ovary (Fig. 3B), suggesting that *EsSoxB2-1* could mainly function in testes. Thereafter we focused on the characterization of its potential role in testis.

### Localization of the *EsSoxB2-1* mRNA and *Es*SOXB2-1 protein in the testes

**Figure 4:**
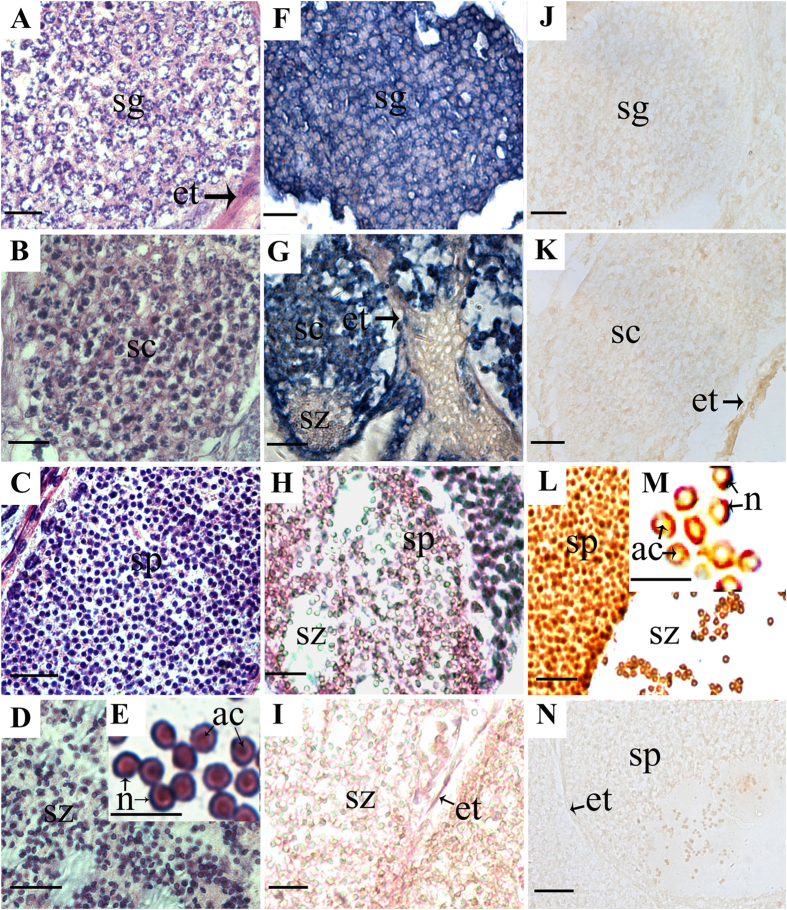
Localization of *EsSoxB2-1* transcripts and *Es*SOXB2-1 proteins in *E. sinensis* testes at various stages: (**A**) Early spermatogenesis (May); (**B**) Developing testis (July), and (**C,D**) Mature testis (September). Regular histological section was stained with hematoxylin and eosin (**A–E**). Transcripts were visualized by *in situ* hybridization with the corresponding DIG-labeled antisense RNA probes (**F–H**) and the sense RNA probes were used as negative control (**I**). The slides were counterstained with neutral red (**H,I**). Proteins were visualized by immunohistochemical detection with the corresponding first antibodies, rabbit anti-*Es*SOXB2-1 (**J–M**). The negative control (**N**) was treated with normal (preimmune) rabbit serum. ac, acrosome; et, epithelia of seminiferous tubules; n, nucleus; sg, spermatogonium; sc, spermatocyte; sp, spermatid; sz, spermatozoa. The scale bar indicates 100 μm.

Like most decapods, the crab testis is formed by numerous seminiferous tubules that contain germ cells at various developmental stages^24^. As shown in Fig. 4, the spermatogenesis process consisted mainly of four developmental stages: (1) spermatogonia, (2) spermatocytes, (3) spermatids and (4) spermatozoa. Spermatogonia were larger in size and each spermatogonium contains a thin rim of cytoplasm around a vesicular nucleus (Fig. 4A). The spermatocytes have an irregularly shaped nucleus that is larger than that of the spermatogonia and is stained by hematoxylin (Fig. 4B), while the cytoplasm is indistinct and acidophilous. Spermatids are smaller and round in shape. Their nuclei show deep stain while the cytoplasm stained grey with eosin (Fig. 4C). In spermiogenesis, the spermatids transform into spermatozoa. An acrosomal vesicle appears in the vicinity of the nucleus and is gradually surrounded by the nucleus. Finally, the cup-shaped nucleus is positioned in the periphery and the acrosomal complex located in the central region of the mature spermatozoa (Fig. 4D,E).

To examine spatio-temporal expression of the RNA and protein of *Es*SOXB2-1 in the crab spermatogenesis, tissue sections of testes in a breeding period were subjected to *in situ* hybridization and immunohistochemical analysis, respectively. When using a DIG-labeled antisense RNA probe, strong signals were detected in early development germ cells including spermatogonium and spermatocyte (Fig. 4F,G), but not in spermatid and spermatozoa (Fig. 4H). No signal was detected in a negative control using a sense RNA probe (Fig. 4I). Contrary to its corresponding mRNA localization, the crab *Es*SOXB2-1 protein was detected only in the nucleus of spermatid and spermatozoa (Fig. 4L,M), whereas no immuno-signal was found in spermatogonia (Fig. 4J) and spermatocyte (Fig. 4K) as in the negative controls (Fig. 4N).

### Subcellular localization of the *Es*SOXB2-1 protein with immunogold labeling

**Figure 5:**
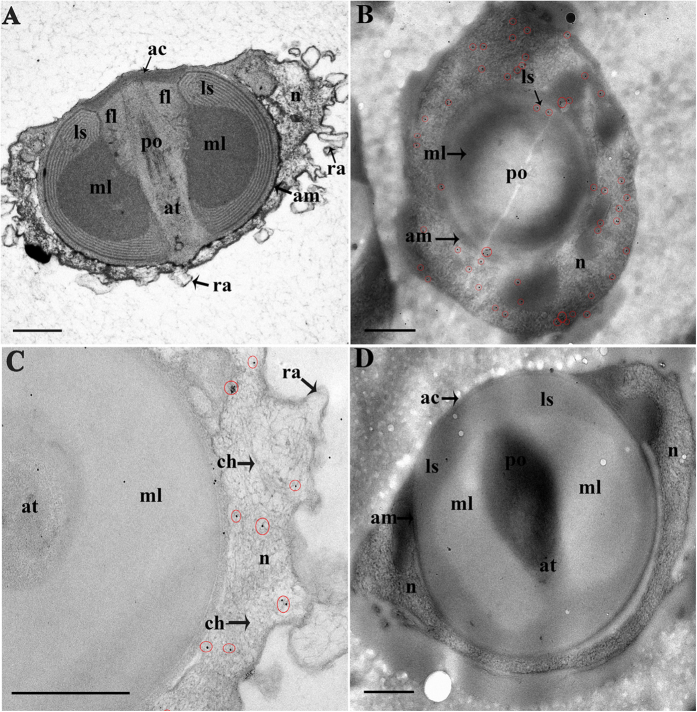
Immunogold electron micrographs of the crab spermatozoa. (**A**) A vertical section with routine staining; (**B,C**) Cross sections stained using rabbit anti-*Es*SOXB2-1 antibodies and colloidal gold-conjugated secondary antibodies; (**D**) Negative control treated with normal (preimmune) rabbit serum. Immunogold signals were highlighted by red circles. The scale bar indicates 0.5 μm. ac, apical cup; am, acrosomal tubule membrance; at, acrosomal tubule; az, acrosome zone; ch, chromatin; fl, fibrous layer; ls, lamellar structure; m, mitochondria; ml, middle layer; n, nucleus; po, percutor organ; ra, radial arm.

To further examine the subcellular distribution of *Es*SOXB2-1 protein in the nucleus of spermatozoa, immuno-electron microscope technic (IEM) was performed. As shown in Fig. 5, the mitten crab spermatozoa are aflagellated. Under electron microscopy, the crab spermatozoon contains a complicated acrosome surrounded by an uncondensed nucleus with radial arms. The acrosomal complex is composed of a lot of subcellula structures like acrosomal tubule, apical cap, acrosomal vesicle, etc (Fig. 5A). In the nucleus, the uncondensed chromatins appear as loose fibrous evenly suspended in the nucleoplasm. The immuno-gold granules of *Es*SOXB2-1 protein were localized on the uncondensed chromatin fibers (Fig. 5B,C). No gold signal was detected in the IEM control section incubated with normal (preimmune) rabbit serum (Fig. 5D).

### Knockdown expression of *EsSoxB2-1* by RNAi

**Figure 6:**
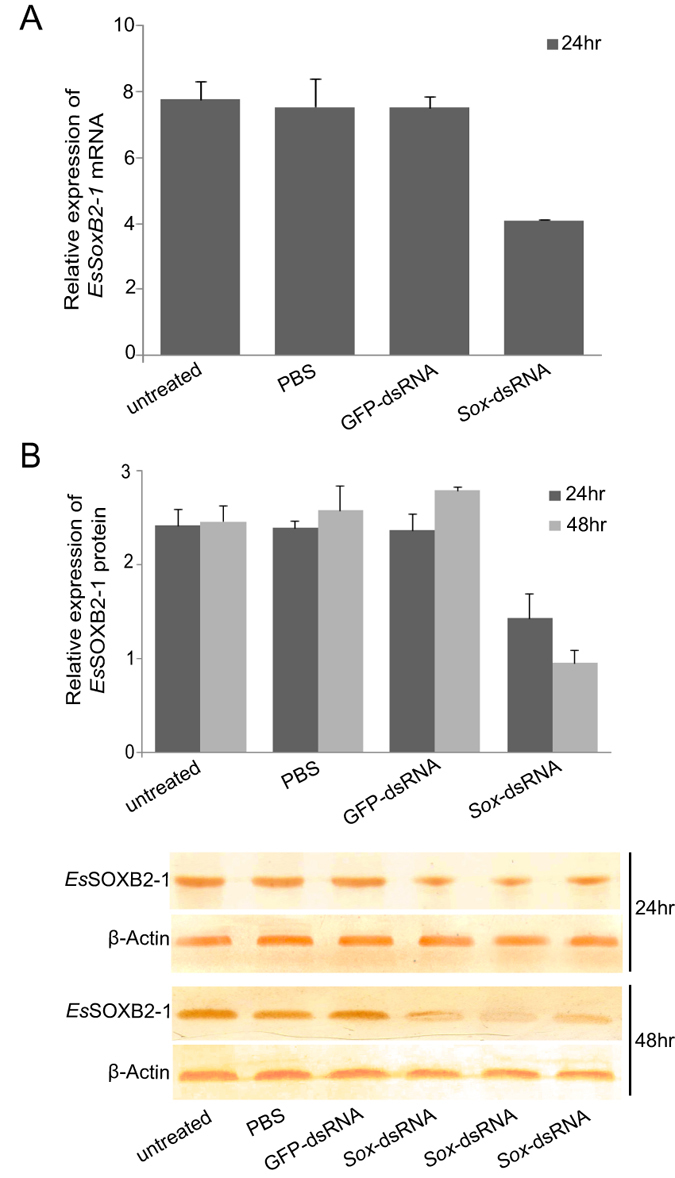
Knockdown effect of *EsSoxB2-1* mediated by RNAi. Expression level of *EsSoxB2-1* mRNA and *Es*SOXB2-1 protein was assayed by qPCR (**A**) and Western blot analysis (**B**), respectively. Three individuals were randomly collected in each group at 24 and 48 hours (hr) post injection of PBS, GFP-dsRNA and *EsSoxB2-1*-dsRNA. The relative expression level was determined using the beta actin as an internal control.

**Figure 7:**
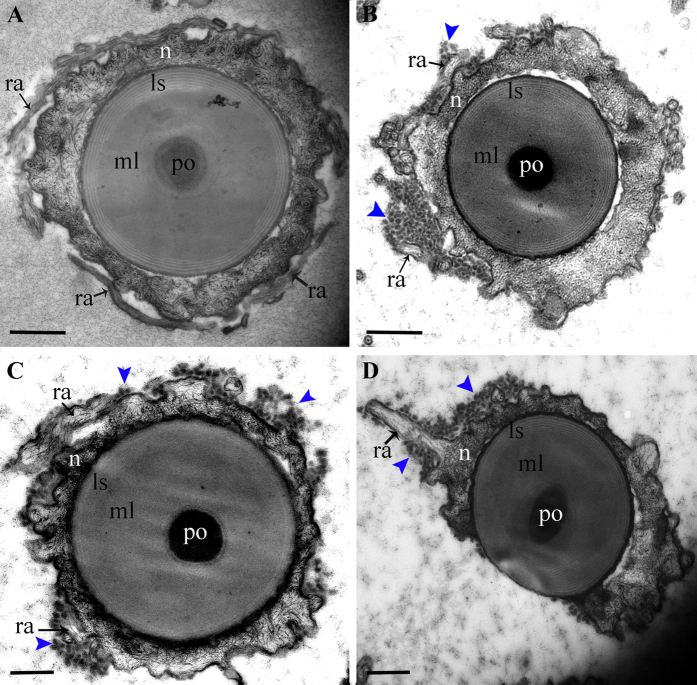
Ultrastructural observation of spermatozoa in PBS group (**A**) and *EsSoxB2-1*-dsRNA group (**B–D**) after one month of repetitive injection. Blue arrow heads point high dense electronic granules generated from the degradation of nucleus arms. The scale bar indicates 0.5 μm. ls, lamellar structure; ml, middle layer; n, nucleus; po, percutor organ; ra, radial arm.

To determine the role of *EsSoxB2-1* in spermiogenesis, RNAi was performed by *in vivo* injection of dsRNA targeting the coding region of *EsSoxB2-1*. The knockdown expression levels of *EsSoxB2-1* in testis were assayed by qPCR and Western blot analysis. At 24 hours post injection, a notably reduction of *EsSoxB2-1* mRNA (about 46%) and protein (about 43%) was detected in *EsSoxB2-1*-dsRNA injected group (Fig. 6A,B), while the expression of *EsSoxB2-1* has no change in control groups after GFP-dsRNA or PBS injection. Subsequently, a lower expression of *Es*SOXB2-1 protein was found at 48 hours post injection of *EsSoxB2-1*-dsRNA (Fig. 6B). The phenotype effects of RNAi knockdown were further observed after *in vivo* repetitive injection for a month. In comparison with normal testicular development in control groups, the size and histological morphology of testes seems to be similar in *EsSoxB2-1*-dsRNA injected group (data not shown). Under transmission electron microscope, however, the mature spermatozoa display abnormal structure. In normal spermatozoa, the cup-shaped nucleus generally extends into radial arms (Fig. 7A), whereas the nucleus arms degraded into many high dense electronic granules after RNAi (Fig.7B–D). Therefore, we concluded that the *EsSoxB2-1* plays an essential role in forming/maintaining nucleus arms of spermatozoa.

Given that histones H3 and H4 were previously revealed to be involved in maintaining uncondensed nucleus^25^, the histones H3 and H4 mRNA levels were additionally assayed at 24 hours and 48 hours post injection. No change was found after knockdown of *EsSoxB2-1* expression (Supplementary Figure S3), indicating that the mRNA expression of histones H3 and H4 is not regulated by *EsSoxB2-1*.

### The detection of *Es*SOXB2-1 protein in spermatozoa during acrosome reaction

**Figure 8:**
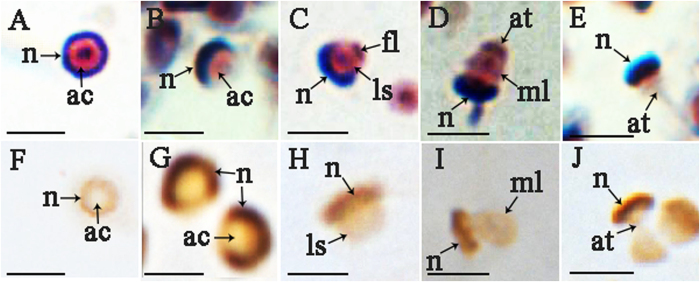
Immunocytochemical detection of *Es*SOXB2-1 protein in spermatozoa during acrosome reaction from step 1 to 4. Regular histological sections were stained with hematoxylin and eosin (**A–E**). Target proteins were visualized with the corresponding first antibodies, rabbit anti-*Es*SOXB2-1 (**F–J**). (**A,F**) unreacted spermatoza; (**B,G**) apical cap protruding (step 1); (**C,H**) acrosomal vesicle valgus (step 2); (**D,I**), extension of the acrosomal tubule (step 3); (**E,J**) acrosomal vesicle dissapearing (step 4). ac, acrosome; ac, spermatozoa evert vesicle; at, acrosomal tubule; fl, fibrous layer; ls, lamellar structure; ml, middle layer; n, nucleus. The scale bar indicates 100 μm.

To investigate whether *Es*SOXB2-1 protein has a potential role in fertilization, *Es*SOXB2-1 protein was traced during the acrosome reaction induced *in vitro* with CaCl2 (Fig. 8A–J). The procedure of acrosome reaction is divided into four typical steps as described by Du *et al.*^26^, namely (1) protrusion of the apical cap (Fig. 8B), (2) eversion of the acrosomal vesicle (Fig. 8C), (3) extension of the acrosomal tubule and contraction of the nuclear cup (Fig. 8D), (4) disappearance of the acrosomal vesicle and the completion of the reaction (Fig. 8E). Immunocytochemistry analysis showed that the immuno-signals of the *Es*SOXB2-1 protein were precisely localized in the nuclear throughout all the four stages of acrosome reaction (Fig. 8G–J), indicating that the *EsSoxB2-1* is not involved in the process of acrosome reaction.

## Discussion

The group B *Sox* genes attract particular interest since they are most closely related to SRY and appear to be functionally conserved during evolution among mammals^2^,^14^. In the present study, we identified a novel *Sox* homolog *EsSoxB2-1* from the mitten crab, *Eriocheir sinensis*. The encoding protein contains a conserved HMG box sharing the highest (about 95%) identity with the beetle *T. castaneum* SOX21b, but there is little similarity in sequence outside the HMG box between them. Further, similar to vertebrate *Sox21*, *EsSoxB2-1* is intronless in the coding region, that is contrary to insect *Sox21* gene with multi-intron structure^14^,^27^. Also, the putative *Es*SOXB2-1 protein contains a polyalanine stretch at C-termini, whereas the polyalanine stretch is absent in the fruit fly *D. melanogaster* SOX21b (Fig. 1A). Unlike vertebrate group B *Sox* genes, however, both the crab *Es*SOXB2-1 and insect SOX21 lack a unique motif for subgroup B members (Fig. 1A). In terms of the gene expression pattern, insects and vertebrates *Sox*21 mainly expressed in nervous system^10^,^22^,^23^,^26^,^28^, whereas the crab *EsSoxB2-1* exhibits gonad-specific expression pattern as revealed by RT-PCR (Fig. 3). Given that insect *Dichaete* also contains no intron^14^, we constructed a phylogenetic tree using full length sequence of SOX proteins, in order to investigate whether *Es*SOXB2-1 is an insect *Dichaete* homolog. The tree showed that *Es*SOXB2-1 first clustered with the beetle *T. castaneum* and the fruit fly *D. melanogaster* SOX21b rather than *Dichaete* (Fig. 1B), implicating that *Es*SOXB2-1 is closely related to insect SOX21b in sequence. Taken together, we concluded that *EsSoxB2-1* is a novel *SoxB2-1* homolog and most likely represents a specific *SoxB2* form of crustacean in the evolution of *Sox* genes.

Spermiogenesis is a highly complicated differentiation process from spermatid to mature spermatozoa. The differentiation of spermatogenic cells appears to be regulated by many nucleus-resident proteins^25^. In vertebrates, haploid spermatids undergo dramatic changes in morphology including reduction of the nuclear size, enlongation of sperm tail and condensation of chromatin. Histones are progressively replaced by protamine to pack genomic DNA, thereby producing more compact chromatin^29^, and along with the removal of cytoplasm^30^. The transcription and translation of many key regulatory genes are switched off to silence all cellular process that are not relevant to fertilization^31^. These sequential changes result in spermatozoa maturation that generates sperm-specific mobility and fertility. Contrary to most species sperm, crab sperm is typically aflagellated and immotile, containing a spherical acrosome surrounded by the uncondensed nucleus. The nucleus of a mature sperm keeps the similar size with spermatids. Spermatid differentiation is characterized by chromatin decondensation. During chromatin decondensation, most histones are reduction and only small amounts of histones H2B and H3 remain in the nucleus of mature spermatozoa in the blue swimming crab *Portunus pelagicus*, which could lead to the disruption of nucleosomal organization and consequently the decondensation of sperm chromatin^26^. However, the molecular mechanism for maturation of the uncondensed sperm nucleus remains unknown in the crab. Our immunocytochemical analysis data showed that *Es*SOXB2-1 protein was not detected in spermatogonia and spermatocyte, but exclusively localized in the nucleus of the crab spermatid and spermatozoa during spermiogenesis (Fig. 4), suggesting involvement of *Es*SOXB2-1 protein in the crab spermiogenesis. To test this hypothesis, we further performed *in vivo* RNAi of *EsSoxB2-1*. Abnormal transformation of the nucleus was observed in the spermiogenesis (Fig. 7), indicating that *EsSoxB2-1* is required for maturation of sperm nucleus. Additionally, our RNAi data showed that the expression of histones H3 and H4 had no significant change after knockdown of *EsSoxB2-1* expression (Supplementary Figure S3), implicating that the mRNA expression of histones H3 and H4 is not regulated by *EsSoxB2-1. EsSoxB2-1* could function as a transcription factor through controlling other protein expression to mediate the movement of histones between nucleus and cytoplasm, although there is no direct interaction between the *EsSoxB2-1* and the histones H3/H4. Further study is needed to identify more RNAi-responsive genes and examine their relationships in sperm nucleus maturation. Intriguingly, several potential SRY/SOX binding sites were identified in the promoter region of *EsSoxB2-1* (Supplementary Figure S2), suggesting *EsSoxB2-1* could be regulated by other *Sox* homologs. Testing this hypothesis would require identifying more SOX protein(s) that can interact specifically with *EsSoxB2-1*. It will be of interesting to further elucidate the regulatory mechanism of *EsSoxB2-1* in spermiogenesis.

Previous studies have shown that the SRY nuclear localization signals (NLSs) are highly conserved during evolution among mammals, and the mutation of NLS can lead to inefficient transportation into the cell nucleus^12^. This means NLSs are essential for translocation of SRY from cytoplasm to nucleus. Like SRY, *Es*SOXB2-1 also has two NLSs at the C- and N-terminal in its HMG domain (Supplementary Figure 1). This may explain why *Es*SOXB2-1 protein display nucleus localization in spermiogenic germ cells during spermiogenesis. Given that some nucleus-resident proteins such as extracellular signal-regulated kinases (ERKs) translocated from the nucleus to the acrosomal tubule during acrosome reaction of the crab spermatozoa^22^,^32^, we extended our studies to trace the *Es*SOXB2-1 protein in acrosome reaction. Interestingly, immunocytochemical analysis showed that *Es*SOXB2-1 protein retained in nuclear without translocation to any other site (Fig. 8). Therefore, we conclude that *Es*SOXB2-1 protein is not involved in acrosome reaction and will remain in the sperm nucleus till fertilization. In fertilization, conventionally, the sperm cell delivered only the paternal haploid genome to the oocyte, which contributed the maternal haploid genome and all the other components required for early zygotic development, such as yolk protein, ooplasm and organelles^33^. However, recent accumulating evidence showed that spermatozoal RNA was present in the zygotic of rat^34^, mouse^35^, and human^36^. Furthermore, the paternal *Wnt4* and *Foxg1* can be translated into protein in zygotes^37^. All these data demonstrated that spermatozoa delivered not only paternal haploid genome to the oocyte but functional mRNA and protein as well when fertilization^37^,^38^. These paternal components include the spermatozoal centriole, transcription factors, and signaling molecules, which are required for early embryonic development^39^. Accordingly, the maintenance *Es*SOXB2-1 protein in the sperm nucleus at the end of acrosome reaction also implicated that *Es*SOXB2-1 protein could be delivered into fertilized eggs along with chromatins functioned as a paternal transcription factor in regulating early embryonic development.

## Conclusion

This study represents the first report on identification and functional characterization of a *Sox* gene in decapod species. Different with known *Sox* B genes in other species, *EsSoxB2-1* has unique gene structure and were found to be specifically expressed in the gonads. The *Es*SOXB2-1 protein is predominantly expressed in the testes and exclusively localized in the nucleus of spermatid and spermatozoa even at the end of acrosome reaction, suggesting that *Es*SOXB2-1 could be delivered into fertilized eggs as the paternal transcript factor in regulating early embryonic development. RNAi knockdown of *EsSoxB2-1* leads to abnormal transformation of the nucleus during spermiogenesis, demonstrating a role for the *EsSoxB2-1* in sperm nucleus maturation.

## Materials and Methods

### Animals and tissues

The mitten crabs were collected from a local fisheries farm. Various tissues, including testis, ovary, heart, muscle, liver, gill and thoracic ganglion, were sampled and immediately frozen in liquid nitrogen and stored at −80 °C until used. Testes were also fixed in Bouin′s fixative (15% saturated picric acid, 5% formalin, and 1% glacial acetic acid) for histological observation. For *in situ* hybridization and immunohistochemical analysis, testes were fixed with 4% paraformaldehyde in phosphatebuffered saline (PBS) solution overnight at 4 °C and stored in methanol at −20 °C after washing three times with PBS. The testis stages were classified as described by Zhang and Qiu^24^.

### Total RNA isolation

Total RNA was isolated from the tissues using TRIzol reagent according to manufacturer’s instruction (Invitrogen, USA). The potential contamination of genomic DNA was excluded by treating with RNase-free DNase I (Promega, USA). The integrity of RNA was assayed by agarose gel electrophoresis and the quantity of RNA was measured by a Nanodrop 2000 spectrophotometer (Nanodrop Technologies, USA).

### Degenerate RT-PCR

A pair of degenerated primers of crab *Sox* gene, sense, (5′-AAGCGACCCATGAA(C\T)GC(A\G\C\T)TT(C\T)AT-3′) and anti-sense (5′-TC(T\C)ACGAGGTCGATA(C\T)TT(A\G)TA(A\G)T-3′), were designed according to the conserved HMG box of different *Sox* genes. A total volume of 25 μL PCR mixture contained 2.5 μL first strand cDNA, 1 μL *Sox* gene sense primer (10 μM), 1 μL *Sox* gene anti-sense primer (10 μM), 2.5 μL 10 × PCR buffer, 1 μL Taq DNA polymerase, 1 μL dNTP (10 mM) and 11 μL ddH_2_O. The PCR cycling parameters included 40 cycles: denatured at 95 °C for 30 s, annealed at 53 °C for 30 s and prolonged at 72 °C for 30 s. The amplified products were purified and ligated into pGM-T vector. The recombinant plasmid was transformed into *Escherichia coli* and positive clones were picked up for sequence.

### Full-length cDNA amplification and sequencing

The full-length cDNA of the crab *Sox* gene was retrieved with 3′ and 5′ RACE method using the Marathon cDNA Amplification Kit (Clontech, USA). The specific primers (5′-GTGGAAGAGTGGCCGCTTGGTGA-3′ for 5′ end amplification and 5′-ATGCTGGAGGATGTGCTGATGGA-3′ for 3′ end amplification) were designed based on the cDNA sequence of the degenerated RT-PCR product. The amplification parameters were 94 °C for 30 s; five cycles of 94 °C for 5 s, 58 °C for 4 min; five cycles of 94 °C for 5 s, 70 °C for 4 min; five cycles of 94 °C for 5 s, 72 °C for 4 min; twenty-five cycles of 94 °C for 5 s, 68 °C for 4 min. RACE products cloned and sequenced were conducted as described above.

### Phylogenetic analysis

The deduced amino acid sequence of the crab *Sox* gene was aligned with those of other metazoan *Sox* genes from GenBank database using the ClustalW (www.ebi.ac.uk/Tools/clustalw2/index.html). A phylogenetic tree was constructed based on the multiple alignment with the neighbor-joining (NJ) method using MEGA 6.06 package^40^.

### Cloning of the genomic sequence of *EsSoxB2-1* and analysis of promoter activity

Genomic DNA was isolated from the crab muscle using phenol extraction method, and the 5′ -flanking region of *EsSoxB2-1* was obtained with the genome walking method using the Universal Genome Walker 2.0 Kit (Clontech, USA). The sequence of 5′ -flanking region was analyzed using the promoter prediction software and the transcription factor binding sites prediction software. Then varying lengths of the 5′ flanking sequences were amplified by PCR using five forward primers F1-F5 and a universal reverse primer at 3′ end (Supplementary Figure S2). The amplicons of the PCR were cloned into the promoterless pGL3-Basic Vector (Promega, USA). Sequence integrity and orientation of cloned inserts were confirmed by sequencing. Transfections to HEK 293T cells were performed using Lipofectamine 2000 Reagent (Invitrogen, USA) when the cells reached about 90% confluence. Cells transfected with the empty pGL3-Basic plasmid are used as a negative control. Cell lysates were harvested 48 hours after transfection, and then promoter activity was assayed by measuring luminescence signal intensity of both the firefly luciferase and sea pansy luciferase using Dual Luciferase Reporter Assay System (Promega, USA). The ratio of firefly luciferase activities and sea pansy luciferase activities were analyzed by one-way ANOVA followed by Tukey’s test using the statistics software SPSS 10.0.

### Tissue distribution of *EsSoxB2-1* mRNA

Tissue distribution of *EsSoxB2-1* mRNA was examined with RT-PCR method. Equal amounts (500 ng) of each total RNA from ovary, testis, thoracic ganglia, heart, liver, muscle and gill were reverse transcribed into first-strand cDNA using M-MLV Reverse Transcriptase (Takara, Japan). Target gene and the reference gene *β*-actin were amplified with gene-specific primers: *Sox*F (5′-CTCCAGAAGAACGGCTACA-3′), *Sox*R (5′-CGCTAGTGAGGTCATGGGT-3′); *β*-actinF (5′-CGACGGTCAGGTCATCACCA-3′) and *β*-actinR (5′-ACGTCGCACTTCATGATGGA-3′). The amplification parameters were 94 °C for 4 min; thirty cycles of 94 °C for 30 s, 54 °C for 30 s, and 72 °C for 1 min; 72 °C for 10 min; stored at 4 °C.

### *In situ* hybridization (ISH)

DIG-labeled cRNA probes were generated from the clone of a 281 bp fragment (position 633–899 bp) of *EsSoxB2-1* cDNA using a DIG RNA Labeling Kit (SP6/T7) (Roche Diagnostics, Germany). The fixed testes tissues were routinely embedded in paraffin and sectioned. *In situ* hybridization on the tissue sections was performed as previously described^41^.

### Preparation of antibody of *Es*SOXB2-1 protein

The open reading frame (ORF) of crab *EsSoxB2-1* gene was cloned into an expression vector pGEX-5X, and then the recombinant plasmid was transformed into BL21 strain. Recombinant *Es*SOXB2-1 protein, obtained from BL21 strain after induced by isopropyl-*β*-d-thiogalactoside (IPTG), was purified through affinity chromatography. The antibody of *Es*SOXB2-1 protein was produced from rabbits as described previously^42^.

### Western blotting and immunocytochemical analysis

The specificity of the antibody and the tissue distribution of *Es*SOXB2-1 protein were determined by Western blotting. Total proteins of testis, heart, muscle, liver, thoracic ganglion, gill were extract, 30 μg of total protein of different tissues were used for SDS-PAGE, proteins in polyacrylamide gel were electrotransfered onto a nitrocellulose membrane, the membrane was treated in blocking solution and incubated with primary antibody overnight at 4 °C followed by a secondary antibody for 2 hours at room temperature. A negative control was set by using the normal (preimmune) rabbit serum. After rinsed in Tris-buffered saline, the membrane was colored in the reagent of DAB Horseradish Peroxidase Color Development Kit (Boster, Wuhan, China). Immunocytochemistry analysis on testis sections was performed as previously described by Qiu *et al.*^41^.

### Immunogold electron microscopy

Mature testes were fixed using 4% paraformaldehyde and 0.5% glutaraldehyde in 0.1 M sodium phosphate and sodium phosphate dibasic buffer (pH 7.05) at 4 °C over night, post-fixed for one hour at 4 °C in 1% osmium tetraoxide, and then embedded in resin and ultrathin-sectioned. The sections were incubated with rabbit anti-*Es*SOXB2-1 protein overnight at 4 °C. For negative control, a normal rabbit serum was used instead of the primary specific antibody. Sections were then incubated with goat anti-rabbit IgG (Sigma, St. Louis, MO, USA) bearing gold particles at 25 °C for 2 hours. Specimens were examined by a transmission electron microscope (JEM-1400, JEOL, Japan).

### Knockdown of *EsSoxB2-1* expression by RNAi

To synthesize dsRNA targeting *EsSoxB2-1*, a 617bp fragment was amplified using specific primers (*EsSox*F4, 5′-tccccgcggTGCCACCAAGAAGGAGGAC-3′ and *EsSox*R4, 5′-ggactagtGCAAGGACGACGCTGACT-3′) and cloned into L4440 plasmid vector with two T7 promotors^43^. Recombinant plasmids were transformed into HT115 (DE3) *E. coli*^44^ to produce sense and antisense RNA of *EsSoxB2-1*. Total RNAs were extracted using TRIzolreagent (Invitrogen, USA), and then heated at 7 °C for 10 min, cooled down slowly to room temperature for annealing. After purified using phenol/chloroform, the resultant *EsSoxB2-1*-dsRNA was assayed using agarose gel electrophoresis, and quantified using a Nanodrop2000c spectrophotometer (Thermo, USA). Meanwhile a green fluorescent protein (GFP) gene was also cloned for production of GFP-dsRNA as a control.

RNAi experiment was performed by *in vivo* injection of dsRNA. Individuals that finished reproductive molting were selected and randomly divided into three groups: *EsSoxB2-1*-dsRNA, GFP-dsRNA and PBS injection groups. Each individual was injected at the base of the fifth pleopods. The injection of *EsSoxB2-1*-dsRNA or GFP-dsRNA was conducted at a concentration of 100 μg in 100 μL of PBS buffer (pH 7.6) per 50 g body weight.

At 24 hours and 48 hours post injection, testis samples from three individuals of each group were collected, frozen in liquid nitrogen and stored at −80 °C. The rest of the crabs of the each group were cultured and repetitively injected every three days for a month with *EsSoxB2-1*-dsRNA, GFP-dsRNA and PBS buffer, respectively. *EsSoxB2-1* mRNA levels was assayed by real-time qPCR using a SYBR Premix Ex Real Time PCR kit (Takara, Japan). The data obtained from real-time PCR were analyzed using the 2^−ΔΔCT^ method and then subjected to one-way analysis of variance (one-way ANOVA) using SPSS statistics software. *Es*SOXB2-1 protein level was assayed by Western blotting. The intensity of target band was quantified using Quantity One software (Bio-Rad, USA) and the significant difference was analysed using SPSS statistics software.

### Induction of acrosome reaction

The spermatozoa were collected from spermatophores in the seminal vesicles. To trace the *EsSoxB2-1* protein in spermatozoa during acrosome reaction, acrosome reaction of the crab spermatozoa was induced by 0.1% CaCl_2._ Samples were collected and fixed using 4% paraformaldehyde in a PBS (pH 7.4) overnight at 4 °C and stored in methanol at −20 °C after washing three times with PBS. The histological observation and immunocytochemical analysis were conducted as above.

## Additional Information

**How to cite this article**: Liu, Z.-Q. *et al.* A novel *SoxB2* gene is required for maturation of sperm nucleus during spermiogenesis in the Chinese mitten crab, *Eriocheir sinensis.* *Sci. Rep.* **6**, 32139; doi: 10.1038/srep32139 (2016).

## Supplementary Material

Supplementary Information

## References

[b1] Sinclair A. H. *et al.* A gene from the human sex-determining region encodes a protein with homology to a conserved DNA-binding motif. Nature 346, 240–244 (1990). 1695712 10.1038/346240a0

[b2] Bowles J., Schepers G. & Koopman P. Phylogeny of the SOX family of developmental transcription factors based on sequence and structural indicators. Developmental Biology 227, 239–255 (2000). 11071752 10.1006/dbio.2000.9883

[b3] Denny P. *et al.* A conserved family of genes related to the testis determining gene, SRY. Nucleic Acids Research 20, 2887 (1992). 1614875 10.1093/nar/20.11.2887 PMC336939

[b4] Harley V. R., Lovell-Badge R. & Goodfellow P. N. Definition of a consensus DNA binding site for SRY. Nucleic Acids Research 22, 1500–1501 (1994). 8190643 10.1093/nar/22.8.1500 PMC308012

[b5] Harley V. R. *et al.* DNA binding activity of recombinant SRY from normal males and XY females. Science 255, 453–456 (1992). 1734522 10.1126/science.1734522

[b6] Pontiggia A. *et al.* Sex-reversing mutations affect the architecture of SRY-DNA complexes. EMBO Journal 13, 6115–6124 (1994). 7813448 10.1002/j.1460-2075.1994.tb06958.x PMC395591

[b7] Koopman P. *et al.* Male development of chromosomally female mice transgenic for Sry. Nature 351, 117–121 (1991). 2030730 10.1038/351117a0

[b8] Wilson M. J. & Dearden P. K. Evolution of the insect Sox genes. BMC Evolutionary Biology 8, 120 (2008). 18439299 10.1186/1471-2148-8-120 PMC2386450

[b9] Schepers G. E., Teasdale R. D. & Koopman P. Twenty pairs of sox: extent, homology, and nomenclature of the mouse and human sox transcription factor gene families. Developmental Cell 3, 167–170 (2002). 12194848 10.1016/s1534-5807(02)00223-x

[b10] Whittington N., Cunningham D. & Casey E. Characterization of the function of Sox21 during Xenopus laevis neural development. Developmental Biology 344, 456–457 (2010).

[b11] Freeman S. D. & Daudet N. Artificial induction of Sox21 regulates sensory cell formation in the embryonic chicken inner ear. PLoS ONE 7 (2012). 10.1371/journal.pone.0046387 PMC3468625 23071561

[b12] Sudbeck P. & Scherer G. Two independent nuclear localization signals are present in the DNA-binding high-mobility group domains of SRY and SOX9. Journal of Biological Chemistry 272, 27848–27852 (1997). 9346931 10.1074/jbc.272.44.27848

[b13] Uchikawa M., Kamachi Y. & Kondoh H. Two distinct subgroups of Group B Sox genes for transcriptional activators and repressors: their expression during embryonic organogenesis of the chicken. Mechanisms of Development 84, 103–120 (1999). 10473124 10.1016/s0925-4773(99)00083-0

[b14] McKimmie C., Woerfel G. & Russell S. Conserved genomic organisation of Group B Sox genes in insects. BMC Genet 6, 26 (2005). 15943880 10.1186/1471-2156-6-26 PMC1166547

[b15] El Jamil A., Kanhoush R., Magre S., Boizet-Bonhoure B. & Penrad-Mobayed M. Sex-Specific Expression of SOX9 During Gonadogenesis in the Amphibian Xenopus tropicalis. Developmental Dynamics 237, 2996–3005 (2008). 18816826 10.1002/dvdy.21692

[b16] Raverot G. *et al.* Sox3 expression in undifferentiated spermatogonia is required for the progression of spermatogenesis. Developmental Biology 283, 215–225 (2005). 15893302 10.1016/j.ydbio.2005.04.013

[b17] Weiss J. *et al.* Sox3 is required for gonadal function, but not sex determination, in males and females. Molecular and Cellular Biology 23, 8084–8091 (2003). 14585968 10.1128/MCB.23.22.8084-8091.2003 PMC262333

[b18] Morais da Silva S. *et al.* Sox9 expression during gonadal development implies a conserved role for the gene in testis differentiation in mammals and birds. Nature Genetics 14, 62–68 (1996). 8782821 10.1038/ng0996-62

[b19] Osaki E. *et al.* Identification of a novel Sry-related gene and its germ cell-specific expression. Nucleic Acids Research 27, 2503–2510 (1999). 10359848 10.1093/nar/27.12.2503 PMC148454

[b20] Rehberg S. *et al.* Sox10 is an active nucleocytoplasmic shuttle protein, and shuttling is crucial for Sox10-mediated transactivation. Molecular and Cellular Biology 22, 5826–5834 (2002). 12138193 10.1128/MCB.22.16.5826-5834.2002 PMC133963

[b21] Smith J. M. & Koopman P. A. The ins and outs of transcriptional control: nucleocytoplasmic shuttling in development and disease. Trends in Genetics 20, 4–8 (2004). 14698613 10.1016/j.tig.2003.11.007

[b22] Cunningham D. D. *et al.* Cloning and developmental expression of the soxB2 genes, sox14 and sox21, during Xenopus laevis embryogenesis. International Journal of Developmental Biology 52, 999–1004 (2008). 18956331 10.1387/ijdb.082586dc PMC2587241

[b23] Richards S. *et al.* The genome of the model beetle and pest Tribolium castaneum. Nature 452, 949–955 (2008). 18362917 10.1038/nature06784

[b24] Zhang E. F. & Qiu G. F. A novel Dmrt gene is specifically expressed in the testis of Chinese mitten crab, *Eriocheir sinensis*. Development Genes and Evolution 220, 151–159 (2010). 20809137 10.1007/s00427-010-0336-2

[b25] Hecht N. B. Gene expression during male germ cell development. In Cell and Molecular Biology of the Testis (eds. Desjardins C. & Ewing L. L.) 400–432 (Oxford University Press, New York, 1993).

[b26] Stewart M. J. *et al.* Spermatogenesis in the blue swimming crab, Portunus pelagicus, and evidence for histones in mature sperm nuclei. Tissue Cell 42, 137–150 (2010). 20413138 10.1016/j.tice.2010.03.002

[b27] McKimmie C., Woerfel G. & Russell S. Conserved genomic organisation of Group B Sox genes in insects. BMC Genetics 6 (2005). 10.1186/1471-2156-6-26 PMC1166547 15943880

[b28] Cunningham D. D. & Casey E. M. S. Function and regulation of Xenopus laevis Sox21. Developmental Biology 319, 497–497 (2008).

[b29] Miller D., Brinkworth M. & Iles D. Paternal DNA packaging in spermatozoa: more than the sum of its parts? DNA, histones, protamines and epigenetics. Reproduction 139, 287–301 (2010). 19759174 10.1530/REP-09-0281

[b30] Nakagawa T., Nabeshima Y. & Yoshida S. Functional identification of the actual and potential stem cell compartments in mouse spermatogenesis. Developmental Biology 12, 195–206 (2007). 10.1016/j.devcel.2007.01.002 17276338

[b31] Sassone-Corsi P. Unique chromatin remodeling and transcriptional regulation in spermatogenesis. Science 296, 2176–2178 (2002). 12077401 10.1126/science.1070963

[b32] Sun W. J. *et al.* ERK is involved in the process of acrosome reaction *in vitro* of the Chinese mitten crab, *Eriocheir sinensis*. Marine Biotechnology (NY) 17, 305–316 (2015). 10.1007/s10126-015-9619-y 25663286

[b33] Meseguer M. *et al.* Effect of sperm DNA fragmentation on pregnancy outcome depends on oocyte quality. Fertil Steril 95, 124–128 (2011). 20643402 10.1016/j.fertnstert.2010.05.055

[b34] Pessot C. A. *et al.* Presence of RNA in the sperm nucleus. Biochemical and Biophysical Research Communications 158, 272–278 (1989). 2463835 10.1016/s0006-291x(89)80208-6

[b35] Wykes S. M., Miller D. & Krawetz S. A. Mammalian spermatozoal mRNAs: tools for the functional analysis of male gametes. Journal of Submicroscopic Cytology and Pathology 32, 77–81 (2000). 10877105

[b36] Wykes S. M., Visscher D. W. & Krawetz S. A. Haploid transcripts persist in mature human spermatozoa. Molecular Human Reproduction 3, 15–19 (1997). 9239704 10.1093/molehr/3.1.15

[b37] Fang P. *et al.* Estimated Diversity of Messenger RNAs in Each Murine Spermatozoa and Their Potential Function During Early Zygotic Development. Biology of Reproduction 90 (2014). 10.1095/biolreprod.114.117788 24671878

[b38] Sutovsky P. & Schatten G. Paternal contributions to the mammalian zygote: fertilization after sperm-egg fusion. International Review of Cytology-a Survey of Cell Biology 195, 1–65 (2000). 10.1016/s0074-7696(08)62703-5 10603574

[b39] Schatten G. The centrosome and its mode of inheritance: the reduction of the centrosome during gametogenesis and its restoration during fertilization. Developmental Biology 165, 299–335 (1994). 7958403 10.1006/dbio.1994.1256

[b40] Tamura K. *et al.* MEGA6: Molecular Evolutionary Genetics Analysis version 6.0. Molecular Biology and Evolution 30, 2725–2729 (2013). 24132122 10.1093/molbev/mst197 PMC3840312

[b41] Qiu G. F., Yamano K. & Unuma T. Cathepsin C transcripts are differentially expressed in the final stages of oocyte maturation in kuruma prawn Marsupenaeus japonicus. Comparative biochemistry and physiology. Part B, Biochemistry & molecular biology 140, 171–181 (2005). 10.1016/j.cbpc.2004.09.027 15649764

[b42] Yang G. C., Cui Z. & Qiu G. F. Prokaryotic expression, antibody preparation of gonad-specific protein *Es*SOX21b-like of the Chinese mitten crab *Eriocheir sinensis*. Biotechnology Bulletin 31, 1–6 (2015).

[b43] Fire A. *et al.* Potent and specific genetic interference by double-stranded RNA in Caenorhabditis elegans. Nature 391, 806–811 (1998). 9486653 10.1038/35888

[b44] Dasgupta S. *et al.* Genetic uncoupling of the dsRNA-binding and RNA cleavage activities of the Escherichia coli endoribonuclease RNase III--the effect of dsRNA binding on gene expression. Molecular Microbiology 28, 629–640 (1998). 9632264 10.1046/j.1365-2958.1998.00828.x

