# Supplementary material for: A novel SoxB2 gene is required for maturation of sperm nucleus during spermiogenesis in the Chinese mitten crab, Eriocheir sinensis
Source: Sci Rep. 2016 Aug 26;6:32139. doi: 10.1038/srep32139 (PMC4999818; doi:10.1038/srep32139)
Supplement: Supplementary Information [file srep32139-s1.pdf]

Title：A novel *SoxB2* gene is required for the maturation of sperm nucleus during spermiogenesis in the Chinese mitten crab, *Eriocheir sinensis*

Authors: Zhi-Qiang Liu[a], Xue-Hui Jiang[a],Hai-Yan Qi, Liang-Wei Xiong, Gao-Feng Qiu*

Key Laboratory of Exploration and Utilization of Aquatic Genetic Resources Certificated by Ministry of Education, College of Fisheries and Life Science, Shanghai Ocean University, 999 Hucheng Huan Road, Shanghai, 201306, P. R. China.

Correspondence: Gao-Feng Qiu, Key Laboratory of Exploration and Utilization of Aquatic Genetic Resources Certificated by Ministry of Education, College of Fisheries and Life Science, Shanghai Ocean University, 999 Hucheng Huan Road, Shanghai, 201306, P. R. China.

TEL: 86-21-61900436; FAX: 86-21-61900436; E-mail: gfqiu@shou.edu.cn

a.The co-first authors

Supplementary Information

1	GCG	GTC	GGC	TCA	CAG	TTC	GCG	ACT	CAG	GCC	GTA	GCG	CGC	GAG	ACA
46	GTG	ACA	GCC	ATG	AAC	CAG	GTT	CCG	TCA	CCA	ACC	ACT	GCG	TCC	CCG
				M	N	Q	V	P	S	P	T	T	A	S	P
91	ACG	GGA	GGT	GCC	ACC	AAG	AAG	GAG	GAC	CAC	ATC	AAA	CGG	CCC	ATG
13	T	G	G	A	T	K	K	E	D	H	I	K	R	P	M
136	AAC	GCC	TTC	ATG	GTC	TGG	TCT	CGC	ATG	CAG	CGG	CGT	AAG	ATT	GCT
28	N	A	F	M	V	W	S	R	M	Q	R	R	K	I	A
181	CAA	GAA	AAC	CCC	AAA	ATG	CAC	AAC	TCC	GAG	ATC	TCC	AAA	CGT	CTG
43	Q	E	N	P	K	M	H	N	S	E	I	S	K	R	L
226	GGC	TCA	GAG	TGG	AAG	CTG	CTG	ACG	GAG	GCC	GAG	AAG	CGT	CCC	TTC
58	G	S	E	W	K	L	L	T	E	A	E	K	R	P	F
271	ATC	GAC	GAG	GCC	AAG	CGT	CTT	CGC	GCC	CAG	CAC	ATG	AAA	GAG	CAC
73	I	D	E	A	K	R	L	R	A	Q	H	M	K	E	H
316	CCG	GAC	TAC	AAG	TAC	CGG	CCT	CGC	AGG	AAG	CCC	AAG	ACG	CTC	CAG
88	P	D	Y	K	Y	R	P	R	R	K	P	K	T	L	G
361	AAG	AAC	GGC	TAC	AGC	TTC	CCC	CTG	CCG	TAC	CTG	GCC	ACC	TCA	GCG
103	K	N	G	Y	S	F	P	L	P	Y	L	A	T	S	A
406	CTG	GAC	CCG	CTC	GGG	CCG	CTC	CAC	CAG	ACC	TAC	TAC	TCC	ACG	CCC
118	L	D	P	L	G	P	L	H	Q	T	Y	Y	S	T	P
451	GCC	GTG	CCC	TCG	CCG	CTC	GAC	GTC	GCG	GGG	GAC	AAG	TCA	CGT	CTC
133	A	V	P	S	P	L	D	V	A	G	D	K	S	R	L
496	TTC	CCC	GGC	GCC	ACG	CTG	CCT	CAC	CAC	TTC	TAC	CCA	AGC	TTT	GAC
148	F	P	G	A	T	L	P	H	H	F	Y	P	S	F	D
541	CCC	CAG	CAC	TTC	AGC	AAG	CTG	GCG	CAG	GAC	CAC	TAC	AAA	CCC	ATG
163	P	Q	H	F	S	K	L	A	Q	D	H	Y	K	P	M
586	ACC	TCA	CTA	GCG	TGT	AAC	GAC	TCC	GCC	GCC	GCC	GCC	GCC	GCC	GCC
178	T	S	L	A	C	N	D	S	A	A	A	A	A	A	A
631	AGC	GTG	TCT	GCC	GCC	TCC	ATG	AGC	GGC	ATG	TCT	ACG	GTG	TCA	GCC
193	S	V	S	A	A	S	M	S	G	M	S	T	V	S	A
676	CTG	TAC	TCC	TCC	CTC	TAC	TCC	AAG	TCA	GCG	TCG	TCC	TTG	CTG	TCG
208	L	Y	S	S	L	Y	S	K	S	A	S	S	L	L	S
721	GGC	ATG	TCG	GCG	GGG	CTG	ACG	GGC	GGG	CAG	CAA	GGC	GCC	CCG	CAA
223	G	M	S	A	G	L	T	G	G	Q	Q	G	A	P	Q
766	CAC	CAG	CTC	TAC	CCA	GGC	TAC	CCG	CCC	TCC	GTG	GAC	CAG	CTG	CGC
238	H	Q	L	Y	P	G	Y	P	P	S	V	D	Q	L	R
811	CGA	CCC	GTC	TCG	GTG	ATT	TTC	TGA	GCC	GAG	TCC	CCG	CCT	CAC	CAC
253	R	P	V	S	V	I	P	★							
856	GAC	ACA	CCG	TAG	TGC	TCA	GCG	TGT	TGT	CAG	TGT	ACA	TAG	TGT	ACA
901	GTA	GTG	AAC	AGT	GGC	CGA	TAT	AAG	CCC	CGA	CGC	TTC	CGT	GGC	AAA
946	AAA	AAA													

Figure S1 The nucleotide and deduced amino acid sequences of the *EsSoxB2-1* cDNA in the Chinese mitten crab *E. sinensis*. The sequences of primers used for degenerated PCR are marked with yellow lines. The sequence of primers used for 5′ RACE-PCR and 3′ RACE-PCR are marked with black lines and red lines, respectively. The grey shaded area highlights the HMG box. Poly-alanine is marked by pink shadow. Four histidine (H) residues were circled and shaded. The NLS is figured with blue lines. And the star denotes stop code.

−1509 CGACGGCCCGGGCTGGTATCATTGATCGCCCGTGTAAACTTCGACAATGTGATACGTATGGATTTCCCGC

Primer F5

−1439 GTGAGGTGAGGCTTTTCCTGTGTTGATACGAGTGACTGGCCGCGGCGCTCCTTCCTGCACCGCCCGTCAA

−1369 GCCGCTGATACGCGACTGTTCTGCCCGCTGACCTGCCAATAACAAAGTGCTATTCTCACCCGCGGCTGTT

−1299 ATTTATACACTCCTCTGAAAATAGAGAAAGCAAGGATATAAATAAAACTTTCTTTTATACATTGTAAACG

−1229 CCGGATACGCTATATTGGTGTTCTAGAGGTGCTTATTAAAGATGTATGATATATTTATTACGTGATATTC

−1159 TTCTTATCCGGTCTCTATAATTCTAAATACTAAAATAGCAGCACGATGCGAGTTACCACCTCAGGACACG

−1089 CAGGTAATCATGGCGAGGCAGGTAAAGGTGTCACGGCCGGGCCCGCGTGTACAGAAACAGGGCCGATGGT

−1019 ATTGATCAGCGTGCACGCCGACAATCGATACTTTACAATAATGTAATGAGCTTCGTTACCTGAAGAAACC

−949 TTTGCCGTTAATGAAAAGTCTTCCTGCATTCATCTGTGTGAGGTCTAATAACCCAAACTAGCAGCAGCCT

−879 CCTTTGGCTCACCCTCTCGAAAACAAGCATTGCGGGTTCAATATTAATATGGTAAGAGCTTCTGCCAGTA

−809 CACCTCCATTAAAAAATTACTTTGCTCTGATGTAACACAGATGAAAAAGCTTTCTTTTGGCACTGTGTTG

−739 GAGCGTTTTGAACACTTCCTACCCTCCAGTTGTCCTCCAGTGTAGAGTGCCGGCACAGAAAGAGGAGGGG

Primer F4

−669 GCCCGATTTTTGCGTTGTTTCGTTCACGCCCCTCAGTCCCCAGGCGGAGGGCGACAAAAGGGGGCTCAGG

−599 GGAGGCTCAGGGCTCAACAAATTATTCAGCCAGTGACAATGAGTGCATAGTGTCAGCTTGGCTTCTGTAG

SRY SOX5 Primer F3

−529 CTTCAGTTTACTTACACACACATCGCGTTATCATTTAGCCGGAGACTAAAAGTGTCGAGGTTCTCTGAGA

−459 GTAACGATCCTTGATCTCTTCCTCTTTTTATTCTGGCATAAAAGTGGCTTTAAGCCCCTCGTAACTTACT

TATA Primer F2

−389 CTGAGGAGAAGGTGCAATTAAAAGTCAGAGAAAAGGTAATGAAAATGGAAAAGCCTCATAGATACTGTCT

SRY

−319 CTTCCACACCAGTGTTGTTTCGCGCCATCAGCAGCCTTCGCTCAGACCTCGTGTCATATTTTCTTGGCGA

SRY

−249 CCTACACATGTGGCTCCGTCTTGGGCCAATCAGCTGGGTCGAGTAGCTGGCACATTTGTAACGGCGACTG

CAAT SRY Primer F1

−179 CCATTGGCTAAGAAAATTGGCGTTGCTCCGCCCTTCTTACTCAGCGATGATTTACCGGCGCGCTGGTTGG

SRY GATA-1 CREB CAAT

−109 CTGCGGGGAAGCTGGGCGTGGTCAGCGGGCGGCTCGGGGGCGCACACTCACCGCGCCACCTCTGTGGTGT

SRY +1 GATA-1 E2F

−39 AATATTACACTTGCCGCCGCACCGTCCATCATTCTTTTTGCGGTCGGCTCACAGTTCGCGACTCAGGCCG

GATA-1 SOX5 +88

32 TAGCGCGCGAGACAGTGACAGCCATGAACCAGGTTCCGTCACCAACCACTGCGTCCC

Primer R

Figure S2 Nucleotide sequence of 5'-flanking region of *EsSoxB2-1*. Nucleotides were numbered from the first nucleotide of the transcription initiation site. The putative *cis*-acting elements, CpG island and ORF region of *EsSoxB2-1* are indicated by black, red and green underlines, respectively. Two basal core promoters are indicated by blue shading. Pink shades highlight one reverse primer (R) and five forward primers (F1-F5) used in amplifying various lengths of 5' flanking regions for activity assay.

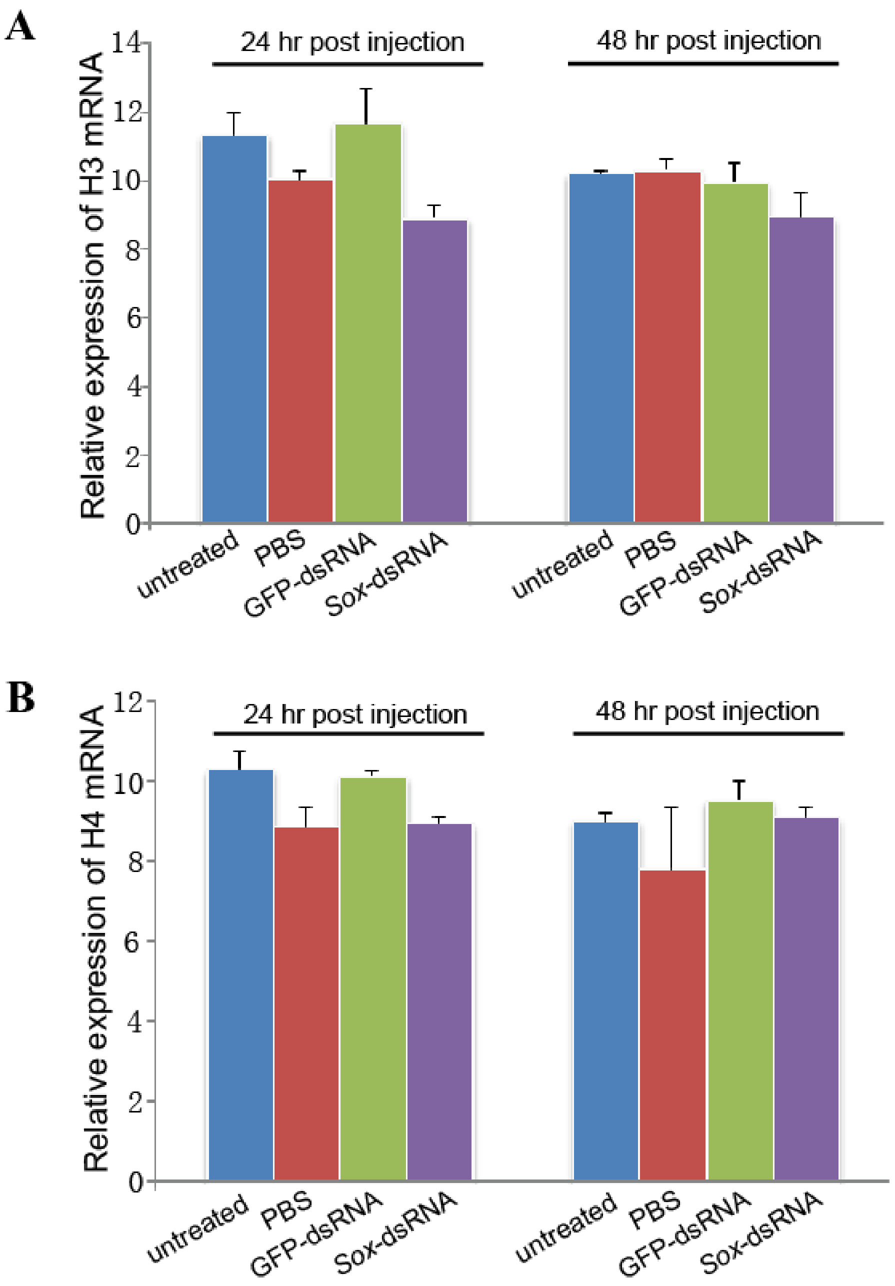

Figure S3 Quantification by qPCR of histones H3 (A) and H4 mRNA (B) in the crab testes at 24 and 48 hours (hr) post injection of *EsSoxB2-1*-dsRNA.

Table S1 List of SOX proteins used in phylogenetic analysis

Species	SOX name	GenBank accession number
Mus musculus	SRY	AAB60446.1
	SOX2	AAC31791.1
	SOX7	P40646.2
	SOX9	NP_035578.3
	SOX13	BAA25786.1
	SOX15	AAF72108.1
Danio rerio	SOX2	BAE48583.1
	SOX4	CAE18168.1
	SOX6	NP_001116481.1
	SOX8	AAX73357.1
	SOX18	XP_001337702.1
	SOX21a	NP_571361.1
	SOX21b	AAS47833.1
Bactrocera dorsalis	SOX5	JAC52640.1
Homo sapiens	SOX3	CAA50465.1
	SOX10	CAG30470.1
	SOX20	NP_008873.1
	SOX21	AAC95381.1
	SOX22	AAB69627.1
Drosophila melanogaster	SOX100b	AAF57112.2
	SOXN	NP_001260269.1
	Dichaete	NP_001261832.1
	SOX21a	AGB94521.1
	SOX21b	AAF49756.1
Xenopus laevis	SOX1	BAE72677.1
	SOX2	NP_001081691.1
	SOX11	AAH70707.1
	SOX12	NP_001079045.1
	SOX17	NP_001081631.1
	SOX21	NP_001165684.1
Oncorhynchus mykiss	SOX24	NP_001117655.1
Gallus gallus	SOX1	BAA25092.1
	SOX2	BAC67545.1
	SOX14	BAA77265.1
Poecilia formosa	SOX1	XP_007565413.1
Thamnophis sirtalis	SOX3	XP_013930026.1
Nipponia nippon	SOX3	XP_009466385.1
Tribolium castaneum	SOXN	EFA04654.1
	Dichaete	EFA04577.1
	SOX21a	EFA04579.1
	SOX21b	EFA04578.1
Zootermopsis nevadensi	Dichaete	KDR12611.1
Takifugu rubripes	SOX14a	AAQ18498.1
	SOX14b	AAQ18499.1
	SOX21	AAQ18500.1
Xenopus tropicalis	SOX14	NP_001093703.1
